# Found through Two Dreams

**Published:** 1888-03

**Authors:** 


### FOUND THROUGH TWO DREAMS.

Some years ago, Dexter, Lambert & Co., the silk mill owners of Paterson, had a foreman in their mill named Peter Turner. He was a man of eccentric habits, very taciturn, and never informed even his wife and family of his business affairs. One day he went to Clifton, two or three miles below Paterson. He started back home after dark, walking on the Erie Railway track. When near Paterson he was struck by the locomotive of a freight train and instantly killed.

A few days before his death Turner had received between $400 and $500 from Dexter, Lambert & Co. Only a few dollars were found on his person when his dead body was picked up, and he had given his wife but $15. What disposition he had made of the balance of the money he had received no one knew. He was not a man of dissolute or extravagant habits, and had been the recipient of a large salary for years, yet after his funeral expenses were paid his family were left penniless. That Turner must have had considerable money deposited, or invested somewhere was certain, but no trace of it could be found.

James Howarth was a near neighbor of the Turners and a close friend of the dead man. A week or so after Turner's death, Howarth went to Mrs. Turner one morning and told her that her late husband had appeared to him in a dream the night before and made a singular revelation.

"My family is in need of some money," the apparition had said, according to Howarth, "and I will supply them with enough to help them over their need for a while. If they will go to the cellar in their house they will find, under a pile of rubbish, a tin blacking box. In that there is $150."

Howarth said that then Turner's apparition disappeared. A search was made as directed, and the tin blacking box and $150 were found. A further search of the cellar and premises were made in hopes that more money might be discovered, but without success. A month or so later Howarth had another dream, in which Turner appeared to him again, and directed him to dig in one corner of the garden, where he would find $100 in a paper package. This money was also found. These two sums was all that were ever discovered of Turner's savings. Howarth's dreams made a great sensation in the city, and some suspicious persons interpreted them in a way that reflected somewhat on Howarth, and gave the impression that he had a knowledge of the entire amount of the Turner money which was not given him by dreams; but that was nonsense, for Howarth was an honest and unassuming mechanic, incapable of such trickery. Besides, if he was aware of the disposition Turner had made of his money and could take advantage of it to his own benefit, why should he have voluntarily despoiled himself of $250 of it by revealing its whereabouts to Turner's family.—*New York Sun.*

